# Elementary School Children Contribute to Environmental Research as Citizen Scientists

**DOI:** 10.1371/journal.pone.0143229

**Published:** 2015-11-18

**Authors:** Victoria L. Miczajka, Alexandra-Maria Klein, Gesine Pufal

**Affiliations:** Institute of Ecology, Leuphana University of Lüneburg, Scharnhorststrasse 1, 21335, Lüneburg, Germany; University of Bologna, ITALY

## Abstract

Research benefits increasingly from valuable contributions by citizen scientists. Mostly, participating adults investigate specific species, ecosystems or phenology to address conservation issues, but ecosystem functions supporting ecosystem health are rarely addressed and other demographic groups rarely involved. As part of a project investigating seed predation and dispersal as ecosystem functions along an urban-rural gradient, we tested whether elementary school children can contribute to the project as citizen scientists. Specifically, we compared data estimating vegetation cover, measuring vegetation height and counting seeds from a seed removal experiment, that were collected by children and scientists in schoolyards. Children counted seeds similarly to scientists but under- or overestimated vegetation cover and measured different heights. We conclude that children can be involved as citizen scientists in research projects according to their skill level. However, more sophisticated tasks require specific training to become familiarized with scientific experiments and the development of needed skills and methods.

## Introduction

Worldwide, ecosystems change rapidly due to human actions and it is therefore vital to understand underlying ecological processes and functions to halt biodiversity loss [1]. Consequently conservation efforts need to be enhanced [2]. Conveying knowledge of biodiversity and ecosystem functions to the public requires new forms of communication between different structures of society [3,4]. To make the importance of conserving the environment and its species and functions more tangible for society, the knowledge transfer should focus on visible ecosystem functions like herbivory, pollination, seed predation and dispersal [5–7]. Citizen science programs that integrate non-professional volunteers into authentic scientific research and conservation efforts, offer therefore an opportunity to promote public engagement and advance research in ecology and conservation by connecting science and education [8]. Furthermore, citizen science projects range from large scales to local research experiences [9] where participants gather valuable data on temporal and geographical variation in monarch butterfly eggs and larvae [10], monitor endangered, threatened and rare plant species in the greater Chicago, Illinois, region [11] or practice sustainable coffee production that comes with sustainable livelihoods [12]. These collections vary in extent, effort, and quality and include different data types like count [13], phenological [14], and species abundance data [15] or estimations on vegetation surveys [16]. Depending on the data type, the collection requires specific skills, time and prior ecological knowledge of the participants. Hence, different studies discuss diverse methods to evaluate the quality and comparability of data collected by citizen scientists with those by scientists [10,15,17,18].

So far, most citizen science projects in conservation ecology concentrate on a specific species (i.e. the endangered Grevy’s zebra (*Equus grevyi*) [19]), a particular region (i.e. the Oak Creek Wildlife Area [20]) or a unique ecosystem (i.e. the Florida Lakewatch [21]). Consequently, participants learn about the target species or a particular interaction between an animal and its habitat [22,23], but more complex interactions or ecosystem functions are rarely approached by citizen scientists.

To assess the land use impact on ecosystem functions in anthropogenic landscapes, we conducted a transdisciplinary research project by combining research on seed predation and dispersal as ecosystem functions with environmental education and the integration of elementary school children as citizen scientists. Studies show that being engaged in ecological experiments together with scientists can increase the children’s knowledge about science in general and ecology specifically [24–26]. In the experimental part of our project, children and scientists investigated seed predation and dispersal by ground-dwelling animals in a cafeteria experiment, because there is little knowledge about these functions along an urban-rural gradient [27]. We chose these functions because they are easy to comprehend, their impact is instantly visible and similar approaches might be transferable to projects on herbivory or pollination. The children had the opportunity to further develop the understanding of their daily surrounding environment, strengthen their systematic thinking early in the process of scientific literacy learning, and be part of authentic scientific research [28–30].

The aim of this case study was to test whether elementary school children were able to conduct an ecological experiment and collect data qualitatively similar to scientists. Specifically, we compared estimated vegetation cover, measured vegetation height and count data for seed removal. We hypothesized that children would achieve similar results to scientists for measured and count data but might over- or underestimate vegetation cover due to their inexperience.

## Methods

### Study area and sites

**Fig 1:**
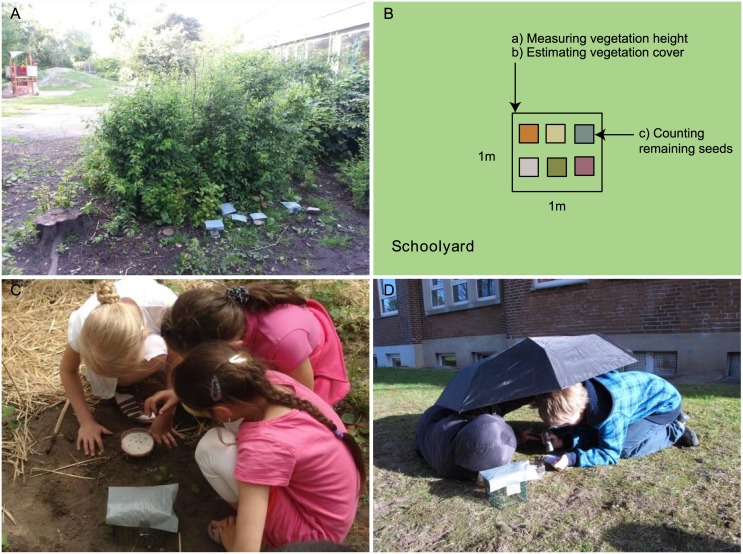
Illustration of the data collection on schoolyards. (A) Experimental set-up at a typical school yard, (B) schematic overview of the experiment with the different tasks children had to carry out, (C) children counting seeds in the treatment and (D) seed counting with UV-flashlights and umbrella.

The study took place along an urban-rural gradient in ten schoolyards in the cities of Hamburg (53°N, 9°E), Lüneburg (53°N, 10°E) and the surrounding area in Lower Saxony, Northern Germany. The structural composition of the schoolyards varied in details but mainly comprised surfaces that were paved, mulched, had bare soil, grassy areas or maintained lawn. All schoolyards had solitary trees, shrubs and hedges and playground equipment (Fig 1A). The seed removal experiment was generally set up on grassy areas and comprised six treatments.

### Educational program

A total of 302 elementary school children (eight to ten years old) from 14 classes in ten schools participated in 12 lessons provided by scientists in each class (S1 Appendix). Each class was taught by one scientist, whereas the teacher only had a supervising role. The scientific and educational content of the project contributed to the official curriculum for grades two to four in Lower Saxony and Hamburg, hence the study was conducted as part of the educational curriculum from the children in school [31,32]. The aims of those core curricula for the children are to formulate questions and test hypotheses. They should also be able to observe and explain natural phenomena, combine the learned knowledge and be able to understand them with the help of observations, questions, descriptions, analyses, measurements and experiments. We supported these by focusing on the concepts of habitats, native and non-native species and plant-animal interactions (specifically seed predation and dispersal) as well as the development of hypotheses and how to answer them. These topics were taught with different interactive exercises, games, discussions and learning materials. During the lessons, children kept a project journal with given exercises and options to document their project participation.

Four lessons were dedicated to the citizen science experiment. Here, we explained the relevance of the children’s participation for our research, children practiced the required tasks (how to fill out the field protocol, setting up the experiment, which variables to count, estimate and measure and how to describe the vegetation) and we discussed the observed data.

Necessary permits to conduct research at the schoolyards and to involve children in the study were obtained from the education authorities in Lower Saxony (Niedersächsische Landesschulbehörde) and Hamburg (Freie und Hansestadt Hamburg, Behörde für Schule und Berufsbildung) in conjunction with the included educational program in schools. Further necessary informed consent from the next of kin, guardians and caretakers on behalf of the children enrolled in the study was given in written form and stored in schools to protect participants’ confidentiality. We did not collect any identifying information about the children. Children taking part in the project only collected empirical data on seed removal so permission from an ethics committee was not required.

### Experimental set-up

Experiments (six treatments total) were set up from April to June 2013 at all sites (see S2 Appendix for specific dates). Each treatment provided access for a specific group of seed removers with different combinations of mesh wire cages, petri dishes filled with sand, plastic rain roofs, bamboo golf trees, insect glue (“Aurum Insektenleim”, Neudorff, Germany) and slug fences (“Snail Stop”,Ringpoint). These combinations allowed access for either slugs, arthropods, earthworms, small rodents, all ground-dwelling seed-removing animals and no animals (control) (comparable set-up see [33]).

Scientists colored seeds with six different water-soluble fluorescent colors (Wicked Colors; CREATEX, USA, airbrush gun 39199 Revell, Germany) [34], with each color corresponding to a specific treatment. Scientists provided ten seeds of oat (*Avena sativa* L.) and ten of red clover (*Trifolium pratense* L.) for each treatment. These seeds were referred to as “big” (*A*. *sativa*) and “small” (*T*. *pratense*), respectively, or their common name, because this was easier to comprehend for the children. We will from now on use *A*. *sativa* and *T*. *pratense*.

A group of two to four children set up one treatment out of six during class in each schoolyard. In seven of the participating classes, treatments were set up simultaneously by all groups (from now on referred to as “simultaneous group”) and one after another in the remaining seven classes (from now on referred to as “sequential group”), depending on the discipline and power of concentration in each class. Consequently, the supervision of the simultaneous group was less intensive than in the sequential group.

### Data collection and comparison

Children used pre-designed field protocols to record: their group members, treatment with its color of seeds, number of exposed seeds, weather conditions, dates of set-up and end of experiment, type of cover (i.e. sand, moss or grass), vegetation cover, vegetation height and number of recovered seeds at the end of the experiment. Vegetation data (cover and height) were assessed in squares of one by one meter by each group at the location of their treatment (Fig 1B), but summarized by the scientists to one data set per class (mean value out of the group data), because some groups filled out their sheet only partly or even lost it (S2 Appendix).

Children recorded cover estimates in words (i.e. “lots of cover”, “without plants”, S2 Appendix) and measured vegetation height with a ruler in centimeter. Scientists estimated vegetation cover in percentages and measured vegetation height at the same times and locations but recorded these data separately. To compare the estimates of vegetation cover from scientists with those of children, vegetation cover was transcribed post-hoc into categories of 25% steps, resulting in five categories (values in between were rounded to the next closest category). Firstly, all phrases used by children to describe vegetation cover were transcribed into the five categories. Hence, “all covered/no free surface with grass, moss, clover” was for example interpreted as 100% of cover or “without plants with sand and soil” was 0% of cover (for the whole transcription see S2 Appendix). Because the sample size of vegetation data was too small for meaningful statistical analyses, we only used the raw data, mean and standard deviation to describe differences in the data between children and scientists (S2 Appendix).

After two nights of exposure, children counted the remaining intact seeds in their treatment and recorded the numbers in their field protocol (Fig 1C). When seeds were missing, they searched for approximately ten minutes to locate potentially dispersed seeds using UV-flashlights (ELECSA 1122; Elecsa, Germany) and black umbrellas to minimize the influence of sunlight (Fig 1D). The missing not detectable seeds were assumed to be predated. Seeds were also classified as predated when they were visibly damaged. Scientists counted seeds in all treatments in the morning of the same day before the children.

For this study, we only analyzed the count data of seeds remaining in the treatments (as a proxy for seed removal) and we did not distinguish between the different treatments when comparing data from children and scientists. We compared seed count data between scientists, simultaneous group and sequential group separately for *A*. *sativa* and *T*. *pratense* seeds. Therefore, we performed two-sided Wilcoxon signed-rank tests for independent groups and non-parametric data for small sample sizes using the software program R [35]. The similarity of data was calculated as the percentage of data points collected by the children that were similar to those of scientists, assuming that the scientists’ data points were all accurate (100%).

## Results

**Fig 2:**
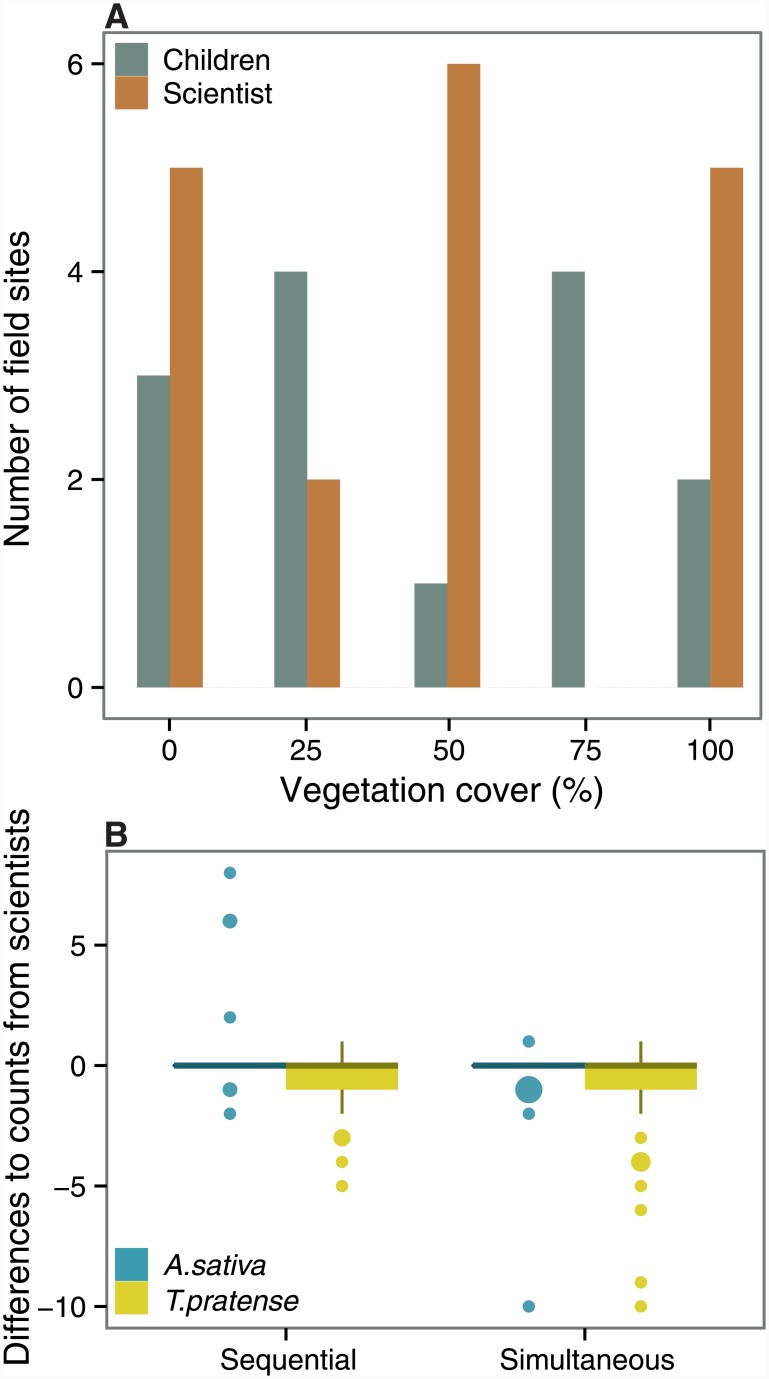
Comparison between estimated vegetation cover and seed count data between children and scientists. Bar plots in (A) show the number of field sites at which the different cover categories (in %) were recorded. Box plots in (B) show the differences of the children’s counts (in sequential and simultaneous groups) compared to seed counts by scientists. The size of the outliers reflects the number of data points.

The vegetation cover recorded by children and scientists ranged from 0% to 100%. Only in five classes out of 14, children and scientists provided similar cover estimates (Table 1, S2 Appendix). Even though mean values of cover estimates are rather similar (Table 1), the direct comparison shows that children either under- or overestimated vegetation cover (Fig 2A, S2 Appendix).

**Table 1:** Comparison of vegetation cover, height and seed count data collected by children and scientists.

Data	Grouping	Mean±SD		Similarity
		Children	Scientists	
Vegetation data				
Cover estimation (%)	class	38.6± 37.7	46.4±40.3	05/14
Height measurement (cm)	class	92.3±225.5	18.9±13.5	01/14
Seed count data				
*A*. *sativa* seeds	Simultaneous group	9.0±2.6	9.4±3.1	78.57%
	Sequential group	9.3±1.9	8.9±2.7	83.33%
*T*. *pratense* seeds	Simultaneous group	7.7±2.2	8.8±2.2	59.52%
	Sequential group	8.2±3.1	9.1±1.8	52.38%

*Note*: Grouping indicates how data was collected by the children (as class, in sequential or simultaneous groups). For vegetation cover and measurement N = 14, for seed count data N = 336 (42 for each group). Similarity is given as matches between children and scientists/field sites for vegetation data and percentages of matching data between children and scientists for seed counts.

Scientists measured vegetation heights between 0–40 cm whereas children measured 5–800 cm (S2 Appendix), corresponding to one matching measurement in one field site out of 14 (Table 1, S2 Appendix).

From the total number of provided seeds (1680), scientists recorded 88.7% remaining in the treatments and children recorded 83.9% seeds. Children in the simultaneous group and scientists counted comparable numbers of remaining *A*. *sativa* seeds (W = 1021, p = 0.090, similarity = 78.57%) and *T*. *pratense* seeds (W = 1057, p = 0.056, similarity = 59.52%) (Fig 2B, Table 1, S3 Appendix). Remaining *A*. *sativa* seeds were also counted similarly by scientists and children in sequential groups (W = 886, p = 0.516, similarity = 83.33%), but in the sequential group children counted significantly fewer *T*. *pratense* seeds (W = 1020, p = 0.042, similarity = 52.38%) compared to scientists (Fig 2B, Table 1).

## Discussion

There was only little concordance in the estimation and measurement of vegetation cover and height data between children and scientists. However, seed count data from children and scientists was mostly similar and differed only considerably when children counted *T*. *pratense* seeds in sequential group.

Prior to our project, the children had no comparable experience or training in conducting scientific experiments. Our results demonstrate that collecting estimates—even using simple phrases—or measuring height is difficult for them. We assumed that describing vegetation cover in words would be sufficient to achieve comparable estimates. However, fractions and percentages are only taught in grade six in secondary school [36] and it appeared that practising a task that the children have no experience in, was not sufficient. The dramatic differences in the measured vegetation height might be due to misinterpretation of the tasks the children were given. Instead of measuring the vegetation height at the location of the experimental set-up, some children also included surrounding shrubs or trees.

Counting is an innate skill for children aged eight to ten because they learn it early in their development [37]. Counting remaining seeds in the treatments therefore resulted mostly in similar data between children and scientists. Children only encountered difficulties in counting *T*. *pratense* seeds (one to two millimeters in diameter), because this task required attention and care and small seeds were missed more often. Even though the difference between sequential and simultaneous groups compared to scientists was small (52.38% vs. 59.52% similarity), this difference was still statistically significant. This is contrary to the assumption that group work is often more effective than whole-class teaching [38]. However, we assume that the differences might be due to an overexcitement from children in sequential groups under the direct supervision of a real scientist and therefore mistakes might have occurred more frequently [39]. Sequential groups were also mostly formed in classes where children were more lively or inattentive. It is therefore difficult to disentangle whether the differences are an inherent effect of the sequential group approach or due to attitudes of the specific children.

We feel confident that our results indicate that it is to some extent possible to integrate elementary school children as citizen scientists in projects that investigate ecosystem functions, if these projects require skills that the children are already familiar with. Citizen science projects that involve skills, which are beyond the children’s educational level, would require intensive preparation and training.

We assume that the children gained a deeper insight into ecological background on native habitats and plant-animal interactions. This did not only support the learning content within the curriculum for grade two to four [31,32], but was also an opportunity to apply and integrate active science learning into their otherwise mostly traditional school routine [40,41]. Our project also gave them the opportunity to take part in actual scientific research and communicate with scientists firsthand about their work, which was shown to be more effective than education by teacher-centered teaching in other studies [24–26,42].

When working not only with children but participants from the public in general, it is important to find the right extent of participation to avoid the risk of losing motivation, which can result in possible errors. This might happen due to overextension and mental over- or underload because participants need time to repeat and practice new methods [43] to gain familiarity with scientific thinking.

It is often criticized that data recorded by citizen scientists is not reliable [15,18], but other approaches show that data recorded by citizen scientists can be qualitatively similar to data collected by researchers [10,23,44–46]. The seed count data from our study were mostly similar between children and scientists, but measuring and estimation data were not, which demonstrates that the reliability of data is task-dependent [17,20]. We therefore emphasize how important it is for citizen science projects to consider the skills and prior knowledge of participants when specific data will be collected. Consequently, it is instrumental to combine experimental approaches with the appropriate educational content to accompany a project, which can benefit scientists and participants alike. We postulate that citizen science projects for children can be designed to a) collect valuable data for ecological research by employing skills that are appropriate for their educational level and to b) support their environmental education in school by addressing topics and methods that are relevant at their specific stage of education. Our study can serve as a blueprint for the development of more transdisciplinary studies to promote public engagement and advance research in conservation programs by connecting science and education early on—by aspiring to make nature conservation more tangible for society and therefore in general more effective, one could not start early enough in childhood to promote nature awareness.

## Supporting Information

S1 Appendix Information on the participating classes (anonymized) and age of children (NA = not available). (DOCX)

S2 Appendix Vegetation data from children and scientists, N = 14 (NA = not available; _a or _b = two classes from the same school took part in the experiment). (DOCX)

S3 Appendix Raw data from the seed experiment from children and scientists, N = 336 (42 for each group), (NA = not available; _a or _b = two classes from the same school took part in the experiment, N original seeds = 10). (DOCX)

## References

[1] Hooper DU, Adair EC, Cardinale BJ, Byrnes JEK, Hungate BA, Matulich KL, et al A global synthesis reveals biodiversity loss as a major driver of ecosystem change. Nature. 2012; 486: 105–10. 10.1038/nature11118 22678289

[2] Carwardine J, O’Connor T, Legge S, Mackey B, Possingham HP, Martin TG. Prioritizing threat management for biodiversity conservation. Conserv Lett. 2012; 5: 196–204.

[3] Bickford D, Posa MRC, Qie L, Campos-Arceiz A, Kudavidanage EP. Science communication for biodiversity conservation. Biol Conserv. 2012; 151: 74–6.

[4] Lindemann-Matthies P, Bose E. How Many Species Are There? Public understanding and awareness of biodiversity in Switzerland. Hum Ecol. 2008; 36: 731–42.

[5] Balvanera P, Siddique I, Dee L, Paquette A, Isbell F, Gonzalez A, et al Linking biodiversity and ecosystem services: Current uncertainties and the necessary next steps. BioScience. 2014; 64: 49–57.

[6] Thomas M, Dawson JC, Goldringer I, Bonneuil C. Seed exchanges, a key to analyze crop diversity dynamics in farmer-led on-farm conservation. Genet Resour Crop Evol. 2011; 58: 321–38.

[7] Tilman D, Reich PB, Isbell F. Biodiversity impacts ecosystem productivity as much as resources, disturbance, or herbivory. Proc Natl Acad Sci U S A. 2012; 109: 10394–7. 10.1073/pnas.1208240109 22689971 PMC3387045

[8] Henderson S. Citizen science comes of age. Front Ecol Environ. 2012; 10: 283–283.

[9] Dickinson J, Shirk J, Bonter D, Bonney R, Crain R, Martin J, et al The current state of citizen science as a tool for ecological research and public engagement. Front Ecol Environ. 2012; 10: 291–7.

[10] Oberhauser K, Prysby M. Citizen science: Creating a research army for conservation. Am Entomol. 2008; 54: 103–5.

[11] Havens K, Vitt P, Masi S. Citizen science on a local scale: The Plants of Concern program. Frontiers in Ecology and the Environment 2012; 10: 321–3.

[12] Chandler M, Bebber DP, Castro S, Lowman MD, Muoria P, Oguge N, et al International citizen science: Making the local global. Front Ecol Environ. 2012; 10: 328–31.

[13] Niven D, Butcher G, Bancroft G. Northward shifts in early winter abundance. Am Birds 2010; The 109TH. 1–6.

[14] Hepper FN. Phenological records of English garden plants in Leeds (Yorkshire) and Richmond (Surrey) from 1946 to 2002. An analysis relating to global warming. Biodivers Conserv. 2003; 12: 2503–20.

[15] Kremen C, Ullman KS, Thorp RW. Evaluating the quality of citizen-scientist data on pollinator communities. Conserv Biol. 2011; 25: 607–17. 10.1111/j.1523-1739.2011.01657.x 21507061

[16] Braschler B. Successfully implementing a citizen-scientist approach to insect monitoring in a resource-poor country. BioScience. 2009; 59: 103–4.

[17] Crall AW, Newman GJ, Stohlgren TJ, Holfelder KA, Graham J, Waller DM. Assessing citizen science data quality: An invasive species case study. Conserv Lett. 2011; 4: 433–42.

[18] Genet KS, Sargent LG. Evaluation of methods and data quality from a volunteer-based amphibian call survey. Wildl Soc Bull. 2003; 31: 703–14.

[19] Low B, Sundaresan SR, Fischhoff IR, Rubenstein DI. Partnering with local communities to identify conservation priorities for endangered Grevy’s zebra. Biol Conserv. 2009; 142: 1548–55.

[20] Galloway A, Tudor M, Haegen W. The reliability of citizen science: A case study of Oregon white oak stand surveys. Wildl Soc Bull. 2006; 34: 1425–9.

[21] Canfield DE, Brown CD, Bachmann RW, Hoyer M V. Volunteer lake monitoring: Testing the reliability of data collected by the Florida LAKEWATCH Program. Lake and Reservoir Management. 2002: 1–9.

[22] Bonney R, Ballard HL, Jordan R, McCallie E, Phillips T, Shirk J, et al Public participation in scientific research: Defining the field and assessing its potential for informal science education. Cent Adv Informal Sci Educ. 2009; 1–58.

[23] Cohn J. Citizen science: Can volunteers do real research? BioScience. 2008; 58: 192–7.

[24] Michael J. Where’s the evidence that active learning works? Adv Physiol Educ. 2006; 30: 159–167. 17108243 10.1152/advan.00053.2006

[25] Benware CA, Deci EL. Quality of learning with an active versus passive motivational set. Am Educ Res J. 1984; 21: 755–765.

[26] Shirk J, Ballard H, Wilderman CC, Phillips T, Wiggins A, Jordan RC, et al Public participation in scientific research: a framework for deliberate design. Ecol Soc Am. 2012; 17: 29.

[27] Türke M, Blattmann T, Knop E, Kindermann A, Prestele J, Marquez L, et al Weeds and endangered herbs have unforeseen dispersal helpers in the agri-environment: Gastropods and earthworms. Renew Agric Food Syst. 2013; 1–4.

[28] Bogner FX. The influence of short-term outdoor ecology education on long-term variables of environmental perspective. J Environ Educ. 1998; 29: 17–29.

[29] Cooper CB, Dickinson J, Phillips T, Bonney R. Citizen science as a tool for conservation in residential ecosystems. Ecol Soc. 2007; 12: 1–11.

[30] Evans C, Abrams E, Reitsma R. The Neighborhood Nestwatch Program: Participant outcomes of a citizen‐science ecological research project. Conserv Biol. 2005; 19: 589–94.

[31] Carstens A, Leißing G, Ommen T, Rahe P, Stein M, Wald A, et al Kerncurriculum für die Grundschule Sachunterricht Niedersachsen. Hrsg vom Niedersächsischen Kultusministerium 2006 1–35. Available: http://db2.nibis.de/1db/cuvo/datei/kc_gs_sachunterricht_nib.pdf

[32] Renz W, Keßler E, Kelpe M, Bischoff M, Michalik K. Bildungsplan Grundschule Sachunterricht, Hamburg. Freie und Hansestadt Hambg Behörde für Schule und Berufsbildung, Landesinstitut für Lehrerbildung und Schulentwicklung, Math Unterricht. 2011; 1–35. Available: http://www.hamburg.de/contentblob/2481914/data/sachunterricht-gs.pdf.

[33] Pufal G, Klein A-M. Post-dispersal seed predation of three grassland species in a plant diversity experiment. J Plant Ecol 2013; 6: 468–79.

[34] Lemke A, von der Lippe M, Kowarik I. New opportunities for an old method: using fluorescent colours to measure seed dispersal. J Appl Ecol. 2009; 46: 1122–8.

[35] R: The R Project for Statistical Computing. 2015. Available: http://www.r-project.org/.

[36] Dönges C, Juraschek B, Kind R, Krüger U-H, Röttger A, Struckmann W. Kerncurriculum für das Gymnasium Mathematik Niedersachsen. 2006; 1–41.

[37] Wynn K. Children's understanding of counting. Cognition. 1990; 36: 155–93. 2225756 10.1016/0010-0277(90)90003-3

[38] Galton M, Hargreaves L, Pell T. Group work and whole-class teaching with 11- to 14-year olds compared. Cambridge J Educ. Routledge. 2009; 39: 119–140.

[39] Izard CE, Schultz D, Fine SE, Youngstorm E, Ackerman BP. Temperament, cognitive ability, emotion knowledge, and adaptive social behavior. Imagin Cogn Pers. 2000; 19: 305–330.

[40] Bouillion LM, Gomez LM. Connecting school and community with science learning: Real world problems and school-community partnerships as contextual scaffolds. J Res Sci Teach. 2001; 38: 878–898.

[41] Prince M. Does Active Learning Work ? A review of the research. J Eng Educ. 2004; 93: 223–231.

[42] Magnuson CS, Starr MF. How early is too early to begin life career planning? The importance of the elementary school years. J Career Dev. 2000; 27: 89–101.

[43] Nissen MJ, Bullemer P. Attentional requirements of learning : Evidence from performance measures. Cogn Psychol. 1987; 19: 1–32.

[44] Danielsen F, Jensen P M, Burgess ND, Altamirano R, Alviola PA, Andrianandrasana H, et al A multicountry assessment of tropical resource monitoring by local communities. BioScience. 2014; 64: 236–51.

[45] Droege S. Just because you paid them doesn’t mean their data are better. Citiz Sci Toolkit Conf. 2007; 1–14.

[46] Schmeller DS, Henry P-Y, Julliard R, Gruber B, Clobert J, Dziock F, et al Advantages of volunteer-based biodiversity monitoring in Europe. Conserv Biol. 2009; 23: 307–16. 10.1111/j.1523-1739.2008.01125.x 19183201

