# Supplementary material for: Elementary School Children Contribute to Environmental Research as Citizen Scientists
Source: PLoS One. 2015 Nov 18;10(11):e0143229. doi: 10.1371/journal.pone.0143229 (PMC4651542; doi:10.1371/journal.pone.0143229)
Supplement: S1 Appendix — (DOCX) [file pone.0143229.s001.docx]

**S1 Appendix. Information on the participating classes (anonymized) and age of children (NA= not available).**

| School | Number of classes | Number of children | Average age of children |
| --- | --- | --- | --- |
| A | 2 | 31 | 7.8 |
| C | 1 | 20 | 9.0 |
| D | 2 | 48 | 9.1 |
| F | 1 | 30 | 8.7 |
| G | 1 | 19 | 8.9 |
| H | 2 | 44 | 9.1 |
| J | 1 | 24 | 9.0 |
| K | 2 | 40 | 9.6 |
| M | 1 | 23 | NA |
| N | 1 | 23 | 9.1 |
| Total | 14 | 302 | 8.9 |
