# Supplementary material for: Elementary School Children Contribute to Environmental Research as Citizen Scientists
Source: PLoS One. 2015 Nov 18;10(11):e0143229. doi: 10.1371/journal.pone.0143229 (PMC4651542; doi:10.1371/journal.pone.0143229)
Supplement: S2 Appendix — (DOCX) [file pone.0143229.s002.docx]

**S2 Appendix. Vegetation data from children and scientists, N=14 (NA= not available; _a or _b = two classes from the same school took part in the experiment).**

| School | Date (2013) | Vegetation cover in % | | Vegetation height in cm | |
| --- | --- | --- | --- | --- | --- |
|  |  | Children* | Scientists | Children | Scientists |
| A_a | May | NA | 25 | 9 | 40 |
| A_b | May | NA | 25 | 15 | 40 |
| C | April | 75 | 100 | 5 | 25 |
| D_a | April | 25 | 0 | 800 | 0 |
| D_b | April | 0 | 0 | NA | 0 |
| F | April | 25 | 0 | 100 | 0 |
| G | June | 0 | 0 | 100 | 15 |
| H_a | June | 25 | 50 | 12 | 30 |
| H_b | June | 25 | 100 | 15 | 30 |
| J | April | 100 | 100 | 20 | 10 |
| K_a | June | 0 | 50 | 6 | 20 |
| K_b | May | 50 | 50 | 5 | 20 |
| M | April | NA | 50 | NA | 15 |
| N | May | 100 | 100 | 20 | 20 |

* qualitative transcription from children’s vegetation cover description (values in between were rounded to the next closest category):

100% ≙ “all covered/no free surface with grass, moss, clover”,

75% ≙“lots of cover/little free surface with plants and sand”,

50% ≙ “half free, half covered”,

25% ≙ “lots of free surface with plants, soil, stones, leaves“,

0% ≙ “without plants, with sand and soil”.
