# Supplementary material for: Elementary School Children Contribute to Environmental Research as Citizen Scientists
Source: PLoS One. 2015 Nov 18;10(11):e0143229. doi: 10.1371/journal.pone.0143229 (PMC4651542; doi:10.1371/journal.pone.0143229)
Supplement: S3 Appendix — (DOCX) [file pone.0143229.s003.docx]

**S3 Appendix. Raw data from the seed experiment from children and scientists, N=336 (42 for each group), (NA= not available; _a or _b = two classes from the same school took part in the experiment, N original seeds=10).**

| School | Plant species | Treatment | Group arrangement | Remaining seeds | |
| --- | --- | --- | --- | --- | --- |
|  |  |  |  | Scientists | Children |
| A_a | *Trifolium pratense* | All | sequential | 10 | 10 |
|  |  | Slugs |  | 10 | 10 |
|  |  | Mice |  | 10 | 10 |
|  |  | Arthropods |  | 10 | 7 |
|  |  | Earthwormm |  | 10 | 8 |
|  |  | Control |  | 6 | 3 |
|  | *Avena sativa* | All |  | 10 | 10 |
|  |  | Slugs |  | 10 | 10 |
|  |  | Mice |  | 10 | 10 |
|  |  | Arthropods |  | 10 | 10 |
|  |  | Earthworm |  | 10 | 9 |
|  |  | Control |  | 10 | 10 |
| A_b | *Trifolium pratense* | All | sequential | 7 | 7 |
|  |  | Slugs |  | 10 | 10 |
|  |  | Mice |  | 10 | 10 |
|  |  | Arthropods |  | 8 | 8 |
|  |  | Earthworm |  | 10 | 10 |
|  |  | Control |  | 10 | 10 |
|  | *Avena sativa* | All |  | 10 | 10 |
|  |  | Slugs |  | 10 | 10 |
|  |  | Mice |  | 10 | 10 |
|  |  | Arthropods |  | 10 | 10 |
|  |  | Earthworm |  | 10 | 10 |
|  |  | Control |  | 10 | 10 |
| C | *Trifolium pratense* | All | sequential | 10 | 8 |
|  |  | Slugs |  | 10 | 10 |
|  |  | Mice |  | 10 | 10 |
|  |  | Arthropods |  | 9 | 8 |
|  |  | Earthworm |  | 10 | 9 |
|  |  | Control |  | 9 | 8 |
|  | *Avena sativa* | All |  | 10 | 10 |
|  |  | Slugs |  | 10 | 10 |
|  |  | Mice |  | 10 | 10 |
|  |  | Arthropods |  | 10 | 10 |
|  |  | Earthworm |  | 10 | 10 |
|  |  | Control |  | 10 | 10 |
| D_a | *Trifolium pratense* | All | simultaneous | 7 | 2 |
|  |  | Slugs |  | 10 | 9 |
|  |  | Mice |  | 8 | 8 |
|  |  | Arthropods |  | 10 | 4 |
|  |  | Earthworm |  | 6 | 6 |
|  |  | Control |  | 9 | 0 |
|  | *Avena sativa* | All |  | 9 | 10 |
|  |  | Slugs |  | 10 | 9 |
|  |  | Mice |  | 10 | 10 |
|  |  | Arthropods |  | 10 | 9 |
|  |  | Earthworm |  | 10 | 10 |
|  |  | Control |  | 10 | 0 |

| School | Plant species | Treatment | Group arrangement | Remaining seeds | |
| --- | --- | --- | --- | --- | --- |
|  |  |  |  | Scientists | Children |
| D_b | *Trifolium pratense* | All | simultaneous | 9 | 9 |
|  |  | Slugs |  | 10 | 10 |
|  |  | Mice |  | 10 | 9 |
|  |  | Arthropods |  | 10 | 10 |
|  |  | Earthworm |  | 8 | 6 |
|  |  | Control |  | 10 | 10 |
|  | *Avena sativa* | All |  | 10 | 10 |
|  |  | Slugs |  | 10 | 10 |
|  |  | Mice |  | 10 | 10 |
|  |  | Arthropods |  | 10 | 10 |
|  |  | Earthworm |  | 10 | 9 |
|  |  | Control |  | 10 | 10 |
| F | *Trifolium pratense* | All | simultaneous | 8 | 9 |
|  |  | Slugs |  | 10 | 10 |
|  |  | Mice |  | 10 | 10 |
|  |  | Arthropods |  | 10 | 9 |
|  |  | Earthworm |  | 10 | 9 |
|  |  | Control |  | 10 | 10 |
|  | *Avena sativa* | All |  | 10 | 10 |
|  |  | Slugs |  | 10 | 10 |
|  |  | Mice |  | 10 | 10 |
|  |  | ArthropodsArthropods |  | 10 | 10 |
|  |  | Earthworm |  | 10 | 10 |
|  |  | Control |  | 10 | 10 |
| G | *Trifolium pratense* | All | simultaneous | 8 | 8 |
|  |  | Slugs |  | 10 | 0 |
|  |  | Mice |  | 9 | 9 |
|  |  | ArthropodsArthropods |  | 9 | 9 |
|  |  | Earthworm |  | 8 | 5 |
|  |  | Control |  | 0 | 0 |
|  | *Avena sativa* | All |  | 0 | 0 |
|  |  | Slugs |  | 10 | 10 |
|  |  | Mice |  | 0 | 0 |
|  |  | Arthropods |  | 10 | 10 |
|  |  | EarthwormEarthworm |  | 10 | 9 |
|  |  | Control |  | 10 | 10 |
| H_a | *Trifolium pratense* | All | sequential | 5 | 0 |
|  |  | Slugs |  | NA | NA |
|  |  | Mice |  | 10 | 10 |
|  |  | Arthropods |  | NA | 1 |
|  |  | EarthwormEarthworm |  | 10 | 10 |
|  |  | Control |  | 9 | 10 |
|  | *Avena sativa* | All |  | 0 | 6 |
|  |  | Slugs |  | NA | NA |
|  |  | Mice |  | 10 | 10 |
|  |  | Arthropods |  | 2 | 4 |
|  |  | Earthworm |  | 0 | 8 |
|  |  | Control |  | 10 | 10 |

| School | Plant species | Treatment | Group arrangement | Remaining seeds | |
| --- | --- | --- | --- | --- | --- |
|  |  |  |  | Scientists | Children |
| H_b | *Trifolium pratense* | All | simultaneous | 6 | 5 |
|  |  | Slugs |  | 10 | 10 |
|  |  | Mice |  | 4 | NA |
|  |  | Arthropods |  | 10 | 10 |
|  |  | Earthworm |  | 9 | 5 |
|  |  | Control |  | 10 | 10 |
|  | *Avena sativa* | All |  | 9 | 9 |
|  |  | Slugs |  | 10 | 10 |
|  |  | Mice |  | 10 | 8 |
|  |  | Arthropods |  | 10 | 10 |
|  |  | Earthworm |  | 8 | 8 |
|  |  | Control |  | 10 | 10 |
| J | *Trifolium pratense* | All | sequential | 9 | 8 |
|  |  | Slugs |  | 9 | 9 |
|  |  | Mice |  | 10 | 10 |
|  |  | Arthropods |  | 10 | 6 |
|  |  | Earthworm |  | 10 | 9 |
|  |  | Control |  | 10 | 9 |
|  | *Avena sativa* | All |  | 2 | 8 |
|  |  | Slugs |  | 10 | 10 |
|  |  | Mice |  | 10 | 10 |
|  |  | Arthropods |  | 10 | 10 |
|  |  | Earthworm |  | 10 | 10 |
|  |  | Control |  | 10 | 9 |
| K_a | *Trifolium pratense* | All | simultaneous | 10 | 10 |
|  |  | Slugs |  | 10 | 9 |
|  |  | Mice |  | 10 | 8 |
|  |  | Arthropods |  | 10 | 10 |
|  |  | Earthworm |  | 10 | 10 |
|  |  | Control |  | 10 | 10 |
|  | *Avena sativa* | All |  | 10 | 10 |
|  |  | Slugs |  | 10 | 10 |
|  |  | Mice |  | 10 | 10 |
|  |  | Arthropods |  | 10 | 10 |
|  |  | Earthworm |  | 10 | 9 |
|  |  | Control |  | 10 | 10 |
| K_b | *Trifolium pratense* | All | sequential | 6 | 5 |
|  |  | Slugs |  | 9 | 8 |
|  |  | Mice |  | 1 | 1 |
|  |  | Arthropods |  | 10 | 10 |
|  |  | Earthworm |  | 7 | 6 |
|  |  | Control |  | 9 | 9 |
|  | *Avena sativa* | All |  | 10 | 8 |
|  |  | Slugs |  | 10 | 10 |
|  |  | Mice |  | 0 | 0 |
|  |  | Arthropods |  | 10 | 10 |
|  |  | Earthworm |  | 10 | 10 |
|  |  | Control |  | 10 | 10 |

| School | Plant species | Treatment | Group arrangement | Remaining seeds | |  |
| --- | --- | --- | --- | --- | --- | --- |
|  |  |  |  | Scientists | Children |  |
| M | *Trifolium pratense* | All | sequential | 10 | 10 | |
|  |  | Slugs |  | 10 | 9 | |
|  |  | Mice |  | 10 | 9 | |
|  |  | Arthropods |  | 10 | 10 | |
|  |  | Earthworm |  | 9 | 9 | |
|  |  | Control |  | 10 | 10 | |
|  | *Avena sativa* | All |  | 10 | 10 | |
|  |  | Slugs |  | 10 | 10 | |
|  |  | Mice |  | 10 | 10 | |
|  |  | Arthropods |  | 10 | 10 | |
|  |  | Earthworm |  | 10 | 10 | |
|  |  | Control |  | 10 | 10 | |
| N | *Trifolium pratense* | All | simultaneous | 10 | 6 | |
|  |  | Slugs |  | 9 | 9 | |
|  |  | Mice |  | 10 | 10 | |
|  |  | Arthropods |  | 10 | 10 | |
|  |  | Earthworm |  | 3 | 3 | |
|  |  | Control |  | 10 | 10 | |
|  | *Avena sativa* | All |  | 8 | 8 | |
|  |  | Slugs |  | 10 | 9 | |
|  |  | Mice |  | 10 | 10 | |
|  |  | Arthropods |  | 10 | 10 | |
|  |  | Earthworm |  | 10 | 10 | |
|  |  | Control |  | 10 | 10 | |
